# Isolation and co-culture of rat parenchymal and non-parenchymal liver cells to evaluate cellular interactions and response

**DOI:** 10.1038/srep25329

**Published:** 2016-05-04

**Authors:** Shyam Sundhar Bale, Sharon Geerts, Rohit Jindal, Martin L. Yarmush

**Affiliations:** 1 Center for Engineering in Medicine, Massachusetts General Hospital, Harvard Medical School and Shriners Hospital for Children, Boston, MA, USA 02114; 2 Department of Biomedical Engineering, Rutgers University, Piscataway, NJ, USA 08854.

## Abstract

The liver is a central organ in the human body, and first line of defense between host and external environment. Liver response to any external perturbation is a collective reaction of resident liver cells. Most of the current *in vitro* liver models focus on hepatocytes, the primary metabolic component, omitting interactions and cues from surrounding environment and non-parenchymal cells (NPCs). Recent studies suggest that contributions of NPCs are vital, particularly in disease conditions, and outcomes of drugs and their metabolites. Along with hepatocytes, NPCs–Kupffer (KC), sinusoidal endothelial (LSEC) and stellate cells (SC) are major cellular components of the liver. Incorporation of primary cells in *in vitro* liver platforms is essential to emulate the functions of the liver, and its overall response. Herein, we isolate individual NPC cell fractions from rat livers and co-culture them in a transwell format incorporating primary rat hepatocytes with LSECs, SCs, and KCs. Our results indicate that the presence and contributions of multiple cells within the co-culture capture the interactions between hepatocytes and NPC, and modulates the responses to inflammatory stimulus such as LPS. The isolation and co-culture methods could provide a stable platform for creating *in vitro* liver models that provide defined functionality beyond hepatocytes alone.

Hepatocyte-based *in vitro* liver models are essential for drug discovery and toxicity screening^1^,^2^,^3^,^4^,^5^. *In vitro* liver models of various complexities, ranging from liver sections, slices, primary hepatocytes, cell-lines, and microfluidic models have been developed to address this goal^1^,^2^,^3^,^5^. Primary hepatocytes form the basic building blocks and are heavily used components in most of these *in vitro* models as they retain most functions after isolation^1^,^2^,^6^,^7^,^8^,^9^,^10^,^11^. Hepatocytes comprise ~80% of the cells in liver, are primarily responsible for drug metabolism, and have a range of functions. However, the liver is a complex unit with multiple cells working together in cohesion, and the contributions of the Non-parenchymal cells (NPCs) are not accounted for in monocultures of hepatocytes^12^,^13^,^14^,^15^,^16^. NPCs, namely Liver Sinusoidal Endothelial (LSEC), stellate (SC) and Kupffer (KC) cells not only provide support for the hepatocytes, but also contribute to inflammatory responses by their own specialized functions within the liver^17^,^18^. LSECs line the hepatocytes and play a significant role in the transport of molecules from the circulating blood to the hepatocytes^1^. SCs are found in the space of Disse and are known to be involved in the formation of Extracellular Matrix (ECM), while KCs provide inflammatory cues and responses to stimuli^1^.

Recent studies have highlighted the importance of NPCs and their contributions to the overall liver response and drug toxicity^8^,^17^,^19^,^20^,^21^. Whole liver and liver slices provide a suitable platform with multiple cells and the architecture in place, however are short lived (few hours to days) and are not optical-friendly making them cumbersome to use for conducting screening studies^22^,^23^. Several studies have focused on isolating^24^,^25^ and incorporating additional NPC components along with hepatocytes: NPC fraction^8^,^9^,^20^,^26^, LSEC^21^,^27^,^28^, KCs^29^,^30^, and SCs^31^,^32^–however it is essential to create co-culture systems with multiple cells in an addressable fashion. While incorporating NPC fractions is a viable option, the use of a mixture cannot be controlled and does not allow the characterization of individual NPCs–while using only one of the NPC cell does not effectively mimic multiple functionality of the liver^8^,^9^,^20^. Isolation of purified non-parenchymal cells and incorporation in *in vitro* models is essential for development of liver models with better functionality^24^,^25^.

Herein, we demonstrate the methods for isolation of primary SCs, LSECs and KC-enriched fraction from rat livers with high purity, incorporation into a transwell format, and culturing upto 7 days. A collagen sandwich culture of hepatocytes was extended to incorporate SCs in a collagen gel on top of hepatocytes, while LSECs and KCs are cultured in a fibronectin coated transwell, forming a (Hepatocyte + Stellate) + (LSEC + Kupffer) co-culture (henceforth referred to as hepatocyte-NPC) system. These co-cultures showed stable albumin secretion and CYP activity, similar to hepatocyte-only cultures. To evaluate the inflammatory response and hepatocyte interaction with NPCs, co-cultures of hepatocyte-NPC, hepatocyte monocultures, and NPC only cultures were prepared and challenged with Lipopolysaccharide (LPS). Hepatocyte-NPC co-cultures showed an LPS-dose dependent TNF-α response, and decrease in CYP activity and albumin production. These results indicate a feedback mechanism from inflammatory factors produced by NPC and its interactions with hepatocytes, causing a decrease in metabolic function, ultimately resulting in reduction of overall hepatic function. We envision these isolation methods and co-culture strategies will provide tools to create multi-cell liver culture models with functions and responses superior than hepatocyte-only models for drug screening and disease models.

## Results

### Isolation of non-parenchymal cells and identification by immunofluorescence staining

**Figure 1:**
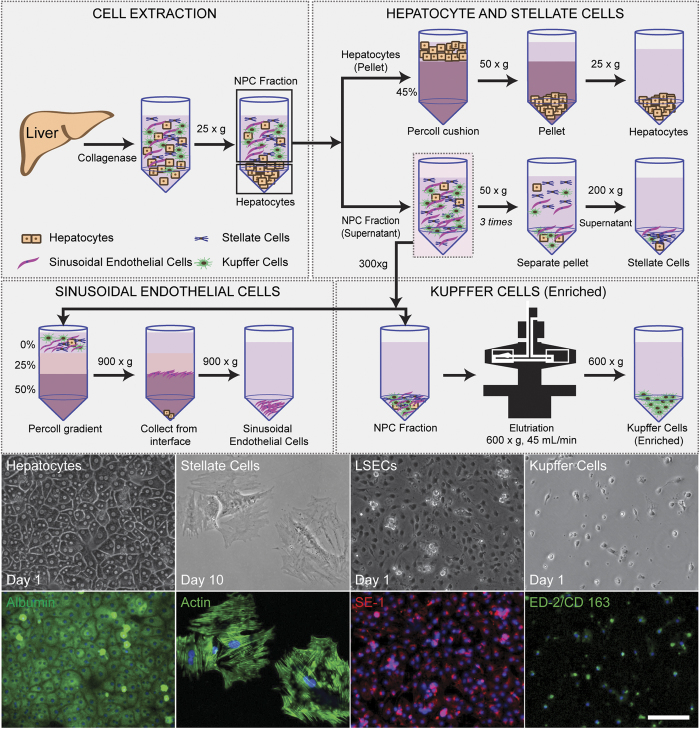
Isolation and characterization of primary hepatocytes and non-parenchymal cell fractions from rat livers. Rat livers were extracted from rats and digested using collagenase and cells obtained as a suspension. Hepatocytes were pelleted and enriched using a percoll cushion (45%), and pelleted. The non-parenchymal fraction is obtained as the supernatant from hepatocyte isolation. Stellate cells were isolated using centrifugation (pelleted at 200 × g) and cultured for 7–10 days with 1 passage before use. Sinusoidal Endothelial cells were isolated using a percoll gradient (interface between 50% and 25%). Kupffer cells were isolated using centrifugal elutriation (at 600 × g, 45 mL/min flow rate) and obtained as a pellet. Isolated cells were stained for markers hepatocytes (albumin), stellate (actin), sinusoidal endothelial cells (SE-1) and Kupffer cells (ED-2/CD 163) respectively. Scale bar = 100 μm.

In this work, we aim to create a primary cell based multi-cellular liver co-culture model from cells obtained from rat livers. To achieve this goal, we have utilized methods for successful isolation of high quality SC, LSEC and KCs from rat NPC fractions and incorporated them with hepatocytes into a co-culture in a layered, addressable fashion similar to the arrangement in the liver. We have adapted reliable and independent methods of isolation, to separate LSECs, SCs and KCs respectively from NPCs (methods, Fig. 1). Briefly, livers were extracted from female Lewis rats and digested using collagenase to obtain a cell suspension containing hepatocytes and NPCs. Following digestion, the hepatocyte fraction was separated using centrifugation and collected as a pellet. Isolated hepatocytes showed high viability and functionality, as seen by albumin staining and albumin secretion in collagen sandwich cultures. The supernatant from post-hepatocyte isolation contained the NPCs, which was separated and used for further isolation of individual NPCs. SCs were separated from NPC fraction by centrifugation^31^ and were maintained in cell culture for 7–10 days before use. Isolated SCs in culture showed a highly organized actin network within the cells, as well as Vitamin A droplets and Desmin (Figure S1). LSECs were isolated from the NPC fraction using a percoll gradient^21^,^33^ and the cells obtained were of high purity (>90%) as determined by SE-1 staining. KC enriched fraction was obtained using centrifugal elutriation^34^ and showed high quality as measured by CD 163 (ED-2) staining and TNF-α response to LPS (Figure S2). Our observations show that these cells were separated with high purity (>90%) and retained cell functionality after isolation. Cell yields were 200–250 M hepatocytes, 0.9–1.2 M SCs, 18–20 M LSECs and 3–5 M KCs per liver isolation.

### Preparation of co-cultures and functional characterization

**Figure 2:**
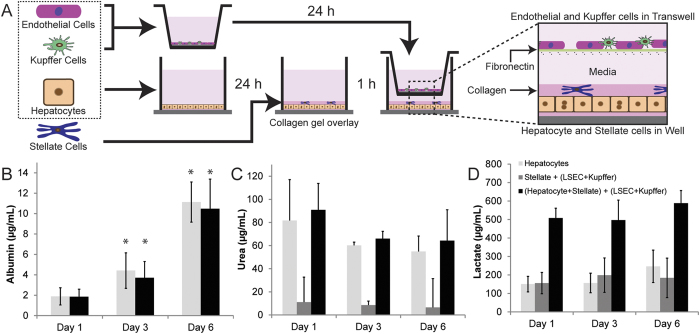
Co-culture setup, and functional activity of hepatocyte monocultures, NPCs and hepatocyte-NPC formats. **(A**) Hepatocytes, LSECs and KCs were isolated from a rat liver and seeded on collagen coated well and fibronectin coated transwell respectively and cultured overnight. SCs are overlaid on top of hepatocytes in a collagen-pre mix and allowed to gel for 1 h. The transwell with LSEC and Kupffer cells was then added to the well to create the co-culture. (**B**) Albumin, (**C**) Urea and (**D**) Lactate production of hepatocyte monocultures, NPC and hepatocyte-NPC cultures. (*p < 0.05, values compared to Day 1).

To create a stratified culture condition using hepatocytes, LSECs, KCs, and SCs, we have extended collagen sandwich culture of hepatocytes to incorporate SCs, with LSECs and KCs cultured in a fibronectin-coated transwell (Fig. 2A). Briefly, hepatocytes were seeded on collagen-coated plates and cultured for 24 hours. Freshly isolated LSECs and KCs (from the same liver for hepatocyte isolation) were seeded onto a fibronectin-coated transwell and cultured for 24 hours. SCs (from a previous isolation and in culture for 7–10 days) were trypsinized, added to a collagen gel pre-mix, and were overlaid on top of the hepatocytes, allowing the gel to form. The transwell containing LSEC and KCs was added on top of the hepatocyte+SC culture in the well. To evaluate the interactions and effect of hepatocytes and NPC in co-culture, control cultures were prepared with A) hepatocyte and B) stellate + (LSEC + Kupffer) cells and were maintained in hepatocyte maintenance media (see methods). In the prepared co-cultures, 0.5 M Hepatocytes, 50,000 SCs, 1 M LSEC and 0.15 M KCs were added with a hepatocyte:SC:LSEC:KC ratio at 10:1:20:3 cells. To assess the function of hepatocytes within the co-cultures, albumin, urea, and lactate production were monitored in the cell culture media. Time-course secretion from various culture configurations on Day 1, 3 and 6 days of co-culture are shown (Fig. 2B–D). Albumin and urea production was similar in hepatocyte and hepatocyte-NPC cultures up to 7 days, while a higher lactate in the cell culture media was observed in hepatocyte-NPC cultures.

### Live/dead microscopy and cellular characterization of co-cultures

**Figure 3:**
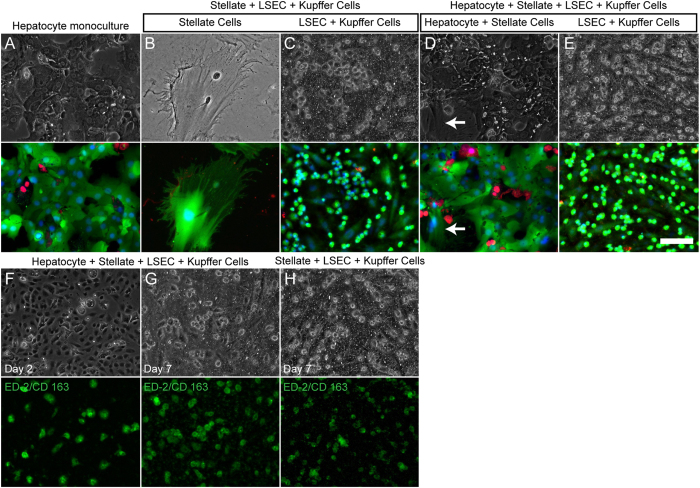
Characterization of cells in co-culture. Phase and live/dead microscopy images of (**A**) Hepatocyte monocultures, (**B**) SCs in NPC, (**C**) (LSEC + Kupffer) in NPC, (**D**) Hepatocyte + SCs in hepatocyte-NPC (arrows point to SC) and (**E**) LSEC + KCs in hepatocyte-NPC cultures on Day 7 of culture. Staining for KCs on (**F**) Day 2, (**G**) Day 7 in hepatocyte-NPC and (**H**) Day 7 in NPC cultures. Scale bar = 100 μm.

Cell viability (calcein AM and ethidium homodimer staining) and morphology of the co-cultures was assessed using fluorescence and phase imaging (Fig. 3A–E). Hepatocyte monocultures within collagen sandwich (Fig. 3A), SCs and LSEC + KCs within NPC cultures (Fig. 3B,C), and hepatocyte + SCs and LSEC + KCs within hepatocyte-NPC cultures (Fig. 3D,E) showed high viability as visualized by calcein staining. To characterize LSECs and KCs within the co-cultures, LSECs and KCs were stained for SE-1 and CD 163 respectively. LSEC + KCs within hepatocyte-NPC showed high expression of SE-1 and CD 163 on Day 2 of co-culture, while LSECs lost significant expression of SE-1 marker by Day 7 (Figs 3F,G and S3). Similarly, LSEC + KCs within NPC cultures showed retention of CD 163 marker on Day 7 of culture while LSECs lost significant expression of SE-1 marker by Day 7 (Figs 3H and S3).

### Inflammatory response of co-cultures to Lipopolysaccharide (LPS) stimulation

**Figure 4:**
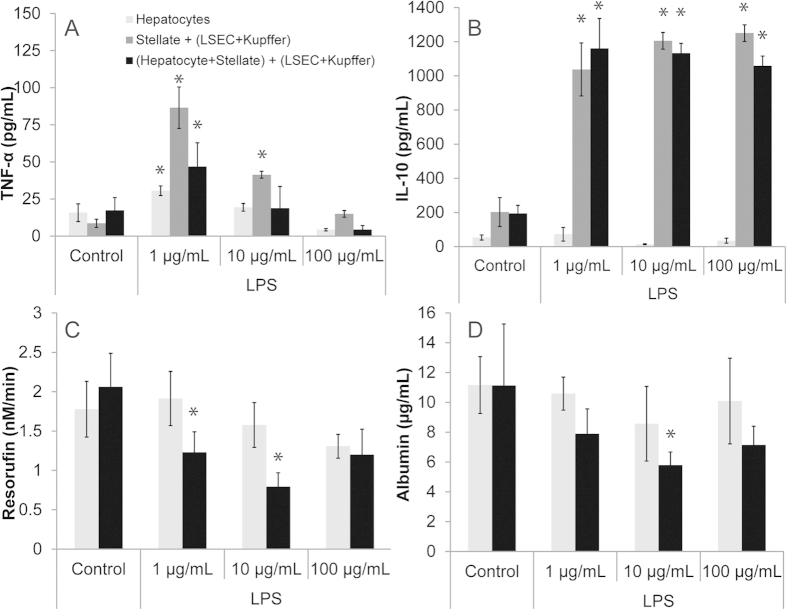
Inflammatory response of hepatocytes, NPCs and hepatocyte-NPCs exposed to Lipopolysaccharide (LPS). (**A**) TNF-α, (**B**) IL-10 secretions (**C**) CYP 1A1/2 Activity and (**D**) Albumin production of Day 6 cultures exposed to LPS for 24 hours. (*p < 0.05, values compared to control).

To evaluate the inflammatory response, we exposed the co-cultures to an inflammatory stimulus (LPS) and measured their response. Co-cultures maintained up to Day 6 were exposed to 0, 1, 10 and 100 μg/mL LPS and their cytokine response and secretion into the media was measured (Fig. 4A,B). In addition, the effect on hepatocyte function in various co-culture formats was evaluated (Fig. 4C,D). Within the hepatocyte-NPC co-culture system, incorporation of multiple cell types leads to a modulated inflammatory response. While hepatocytes monocultures and KCs within NPC show an increase in TNF-α release into the media, the response in hepatocyte-NPC is reduced in comparison with NPC cultures (Fig. 4A). In contrast, KC monocultures have lost their functional response within Day 3 of culture (Figure S2). While the TNF- α response showed a dose and culture-dependent response, IL-10 secretion in both NPC and hepatocyte-NPC cultures was similar (Fig. 4B). Hepatocyte response to LPS exposure, and the effect of secreted inflammatory factors by NPC in hepatocyte-NPC cultures was evaluated using CYP1A1/2 activity and albumin response. We observed a decrease of CYP activity and albumin secretion within hepatocyte-NPC cultures, while no significant response was observed in hepatocyte monocultures (Fig. 4C,D).

## Discussion

Incorporation of NPCs with hepatocytes in *in vitro* models is essential for developing platforms that have applications in drug metabolism and disease models. Herein, we demonstrate the successful isolation of individual non-parenchymal cells from rat livers and the assembly of a liver co-culture model comprising of rat hepatocytes and non-parenchymal cells to mimic the organization within the liver. Our co-culture model (Fig. 2) is based on standard monolayer of hepatocytes cultured in a collagen sandwich, a model which is frequently used in pharmacological industry for drug toxicity screening and mechanistic studies, and which allows for long term culture^6^,^7^,^21^,^35^. Within the rat liver, hepatocytes comprise 60–65%, SCs–8%, LSECs–16%, and KCs–12% cells^14^,^36^,^37^. These cell proportions within the liver indicate a hepatocyte:SC:LSEC:KC ratio of 10:1.3:2.7:2. The hepatocyte-NPC co-culture system in this work incorporates the cells with a ratio 10:1:20:3 such that the relative proportion of hepatocytes, SC, and KC is similar to the ratio *in vivo*. However, our system incorporates greater number of LSECs due to the difficulty associated with maintaining sub-confluent cultures of LSECs. Analysis of cellular secretions indicates that hepatocytes within these co-cultures were stable and retained their functions (Fig. 2). In addition, SCs and KCs showed high viability and retained phenotype in co-culture, while LSECs showed a decrease in SE-1 expression. Culture conditions that promote long term maintenance of LSECs remain challenging. Co-culture with hepatocytes has enabled long term maintenance of LSECs, albeit under specialized condition such as collagen overlay^21^ or short range interaction mediated by hepatocyte/fibroblast^38^. The latter study further demonstrated that culture configuration where LSECs were separated by transwell from hepatocytes or hepatocytes/fibroblast failed to promote maintenance of LSECs. We observed the similar lack of maintenance of LSECs in our model where hepatocytes were separated from LSECs by transwell (Figure S3). Since dedifferentiation of LSECs has been observed under inflammatory microenvironment created by Kupffer cells^39^ and KC secrete TNF-α on isolation (Figure S2), the presence of KC alongside LSECs (in our model) is likely not conducive to maintenance of LSECs.

**Figure 5:**
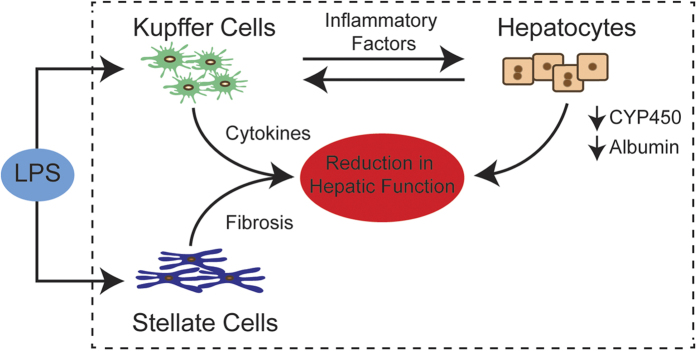
Interactions between hepatocytes and non-parenchymal cells in co-culture. External Stimuli and cellular secretions from non-parenchymal cells influence the behavior and overall outcomes. Capturing cellular interactions and evaluating the cumulative responses are essential to create *in vitro* liver mimics.

While KC monocultures have lost the response to LPS exposure, their functionality is retained to an extent within the co-culture models (NPC and hepatocyte-NPC) (Fig. 3F–H). An interesting observation of the hepatocyte-NPC co-cultures is the mitigated response to LPS, possibly due to the presence of hepatocytes and the interactions with KCs^40^,^41^. While Kupffer cell monocultures maintain their inflammatory response for a relatively short period of time 24–48 hours, their long-term culture is not stable to elicit any responses to inflammatory stimulus^42^. In the co-cultures, we captured a response to LPS exposure on day 6 of culture in which TNF-α secretion by KC is less in hepatocyte-NPC co-culture than in NPC co-culture (Fig. 4A). This could be due to the potential uptake of TNF-α by hepatocytes^43^ through surface receptors resulting in a decrease of TNF-α concentration in hepatocyte-NPC co-culture systems^44^ or hepatocyte mediated reduction in TNF-α secretion by KC. In comparison to primary hepatocyte monocultures that have been shown to secrete relatively low amounts of cytokines and TNF-α upon inflammatory challenge^36^,^42^, which is also observed in our experiments (Fig. 4), incorporation of NPCs enables capture of certain key aspects of the inflammatory response of liver mediated by cytokines. In response to LPS, not only there is secretion of cytokines such as TNF-α and IL-10 in hepatocyte-NPC cultures, but there is also modulation of hepatocyte function as measured by the CYP and albumin activity. Both CYP activity and albumin secretion is decreased post LPS exposure likely due to intercellular interaction occurring between KC, hepatocytes, and the effects of TNF-α and other inflammatory cytokine secretions (Fig. 4C,D). Presently, the hepatocyte-NPC model potentially recapitulated some of the regulatory mechanisms seen *in vivo* within a co-culture system (Fig. 5)^45^,^46^. Although only the effect of co-culturing hepatocytes with KCs and other NPCs in regards to TNF-α and IL-10 secretion was shown, exploring other potential interactions of hepatocytes and NPCs in the co-culture system will allow for further investigative work.

## Conclusion

In summary, our work is a demonstration of the techniques for achieving a multi-cell primary culture of liver cells to mimic cellular arrangement and long-term culture. This manuscript details the isolation of four main liver cell types as well as their assembly; and that the majority of cell populations within the co-culture retain their functionality and stability. While several *in vitro* liver models have been proposed, it is becoming increasingly important to incorporate NPCs into *in vitro* liver models to mimic organ physiology. Such models could lead to increased sensitivity to monitor drug toxicity and function detection with therapeutically relevant concentrations, and in diseased states in particular. For instance, *in vitro* models can be manipulated to provide varied conditions within the liver, such as fibrotic, inflammatory processes that lead to changes in overall hepatic function, which is lost in a hepatocyte-alone model. Taken together, our results demonstrated that the multi-cellular liver model shown here capture aspects of tissue physiology other *in vitro* hepatocyte-alone cell models lack. In future, these models can provide information regarding drug metabolism in both healthy and disease states, which is essential in drug screening process. Multi-cellular co-cultures enable understanding the interplay between various cells, however, further questions about the effect of cell-cell communication and effect of cells within same microenvironment is a matter of future investigation.

## Materials and Methods

### Primary rat hepatocyte isolation

Hepatocytes were obtained from female Lewis rats using two-step collagenase protocol. Two to three month old female Lewis Rats (Charles River Laboratories, Wilmington, MA) weighing 150–175 g were used as a hepatocyte source and were maintained in accordance with National Research Council guidelines. Experimental protocol for cell isolation from rat livers was approved by the subcommittee on research animal care at Massachusetts General Hospital. All procedures were carried out in accordance with the approved guidelines. Using a modification on the two-step collagenase perfusion method^47^,^48^, which involves purification of cell suspension by means of centrifugation over percoll, we routinely isolated approximately 200–300 million (M) hepatocytes per rat liver with 85–90% viability as evaluated by trypan blue exclusion.

### Hepatocyte staining

Hepatocytes were stained for albumin using anti-albumin antibody (abcam #53435). Briefly, freshly isolated rat hepatocytes in culture were fixed in ice-cold Methanol for 20 min followed by incubation with PBS containing 1% BSA and 0.3% Triton-X100 (blocking buffer). Cells were then exposed to 0.2% FITC-anti albumin antibody and DAPI solution in blocking buffer for 1 hour at room temperature, washed with imaging solution and imaged.

### Non-Parenchymal Cell Fraction

The rat non-parenchymal cell (NPC) fraction was obtained as the supernatant from the first centrifugation step performed to pellet primary hepatocytes. Typically, 150–200 M cells were obtained as the NPC fraction per isolation. LSEC and Kupffer cells were obtained from a single isolation, using 60–70% NPC for LSEC and the remainder for Kupffer cell isolation. Stellate cells were obtained from a previous isolation and maintained in culture.

### Stellate cell separation

Stellate cells were separated from NPC fraction using a two-step centrifugation protocol as previously described^31^. Briefly, the NPC fraction was collected in 50 mL conical tubes and centrifuged at 50 × *g* for 5 min, without brake. The supernatant after the spin was collected in fresh conical tubes and the pellet (containing LSEC and Kupffer cells) was collected separately. To increase the purity of the stellate fraction, 5mL of PBS in the bottom along with pellet was discarded for each spin and only the top fraction was collected. The procedure was repeated at least 3 times or until no pellet was observed. The supernatant was then centrifuged at 200 × *g* for 10 min, without brake. The supernatant was removed and the pellet was collected and suspended in DMEM (*for separating the other NPCs from the same isolation, pellets and supernatants were pooled and centrifuged at 300* × *g for 15* *min and used*). The cells were washed by centrifuging at 200 × *g* for 10 min with DMEM. Purified stellate cell fraction was plated in a tissue culture treated T-75 flask and incubated at 37 °C, 5% CO_2_. Media was changed every 24 h for the first 3 days after which cells were trypsinized and re-seeded and maintained in DMEM.

### Stellate cell staining

#### Actin

*S*tellate cells were fixed by incubating with 4% Paraformaldehyde for 10 min at 37 °C followed by incubation with 0.08% Triton X-100 for 10 min at 37 °C. To these cells, a mixture of 1% solution of Alexa 488-Phalloidin and DAPI was added and incubated for 1 hour at 37 °C. Staining solution was replaced with fresh imaging solution and imaged using a fluorescent microscope.

#### Desmin

Stellate Cells were fixed by incubating with 4% Paraformaldehyde for 10 min at 37 °C. To these cells, a mixture of 1% solution of Alexa 488-anti-desmin antibody (in Imaging solution) and DAPI was added and incubated for 1 hour at 37 °C. Staining solution was replaced with fresh imaging solution and imaged using a fluorescent microscope.

#### Lipid droplet staining

Lipid droplets within the cells were visualized staining with BODIPY. Briefly, Stellate cells were fixed by incubating with 4% Paraformaldehyde for 10 min at 37 °C. To these cells, a mixture of 1% solution of BODIPY (in Imaging solution) and DAPI was added and incubated for 1 hour at 37 °C. Staining solution was replaced with fresh imaging solution and imaged using a fluorescent microscope.

### Kupffer cell enriched fraction

Kupffer cell enriched fractions were separated from the NPC fraction using a modified centrifugal elutriation protocol^34^. Briefly, the NPC fraction (in 50 mL conical tubes) was centrifuged at 300 × *g* for 15 min. The supernatant was discarded and the pellet was re-suspended in ice cold PBS. Typically, the NPC fraction from a rat liver was suspended in 20–25 mL PBS (~3–4 × 10^6 ^cells/mL). The cells were then passed through a cell strainer (40 μm pore size) to remove any debris, collected in a syringe, and stored on ice.

#### Setting up the elutriator

All the solutions used for elutriation (Beckman Avanti J25 centrifuge with JE-6.0 rotor) were filter sterilized using a 0.2 μm filter and chilled to 4 °C before use. The temperature of the elutriator was set to 4 °C and sterilized by flowing through solutions in the following order 1) water (45 mL/min, 0 × *g*, 5 min), 2) 6% H_2_O_2_ (10 mL/min, 50 × *g*, 5 min), 3) 15 mg/100mL catalase solution (10 mL/min, 0 × *g*, 5 min), 4) sterile water (10 mL/min, 40 × *g*, 5min), and 5) Hank’s Balanced Salt Solution (HBSS, 10 mL/min, 40 × *g*, 5 min). The cells were separated in HBSS. Care was taken to ensure there were no bubbles formed within the tubing. The elutriator was ramped to 600 × *g* and a flow of 10 mL/min was maintained.

Cell sample was introduced into the elutriator using a syringe connected to the 3-way valve between the buffer reservoir and pump. Briefly, 10–15 mL of the NPC fraction was loaded into a syringe, avoiding any bubble formation. The syringe end of the 3-way valve was sterilized with ethanol and filled with sterile HBSS. The syringe containing the NPC fraction was attached by liquid-liquid contact, avoiding any bubble formation. Once attached, the cells were introduced into the elutriator by switching the source to the syringe, and shutting down the buffer reservoir. 10 mL of the NPC fraction was introduced into the elutriator (at 10 mL/min) and the source was immediately switched to the buffer reservoir. Cells loaded into the elutriator were washed for 10 min to remove any cell debris, while maintaining the rotor at 600 × *g*, and flow at 10 mL/min. Contaminating cells (LSECs and stellate) were washed out at 600 × *g*, 22.5 mL/min. Kupffer cells were eluted at 45 mL/min, 600 × *g* and 100 mL of the cell suspension was collected and stored on ice. The cells were pelleted at 500 × *g* for 7 min (no brake), re-suspended in fresh hepatocyte maintenance media and used as an enriched fraction.

#### Kupffer cell staining

Kupffer Cells were identified using CD 163 (ED-2) Antibody. Briefly, Kupffer cells were incubated with a PBS containing 0.2% Alexa-488 CD 163 antibody and DAPI solution for 1 hour at 37 °C. Staining solution was replaced with fresh imaging solution and imaged using a fluorescent microscope.

#### Inflammatory response of Kupffer Cells

Kupffer cell enriched fraction was seeded on a fibronectin coated (50 μg/mL) 24 well plate at 0.15 M cells/well in 500 μL hepatocyte maintenance media and allowed to attach overnight. To the cells, 1 μg/mL Lipopolysaccharide (LPS) was added and maintained for 24 hours. At the end of incubation, media was collected and the TNF-α content in the media was measured.

### Liver Sinusoidal Endothelial Cell Separation

Liver Sinusoidal Endothelial Cells (LSECs) were separated from NPCs using density separation in a percoll gradient^21^,^33^. Briefly, NPC fraction in 50 mL conical tubes was centrifuged at 300 × *g* for 15 min. The supernatant was discarded and the pellet was suspended in ice cold PBS. Typically, the NPC fraction from a rat was suspended in 20–30 mL ice cold PBS.

#### Percoll Gradient

A percoll gradient was prepared in 50 mL conical tubes with 15 mL of 50% percoll on the bottom and 20 mL of 25% percoll layered on top of the first percoll layer. 10 mL of the NPC fraction was carefully placed on the 25% percoll layer and centrifuged at 900 × *g* for 25 min, without brake. At the end of the centrifugation, the layer between 10–17.5 mL of the percoll gradient is collected and diluted in ice cold PBS (to 50 mL) and centrifuged at 900 × *g* for 25 min without brake to pellet the cells. Any remaining percoll was aspirated and the cells were suspended in fresh hepatocyte culture media (10 mL). To remove any contaminating cells (Kupffer), the cell fraction was incubated on a 10 cm diameter tissue culture dish for 1–2 min and the non-adherent cells were collected. The tissue culture plate was discarded.

#### Endothelial cell staining

Endothelial cells were identified by staining for SE-1. Briefly, cells were washed with fresh media and incubated with a solution of 1:1000 dilution of Hepatic Sinusoidal Endothelial Cells Antibody (SE-1) [DyLight 550] (http://www.novusbio.com) and DAPI stain for 1h at 37 °C. Samples were washed in imaging Solution and imaged using a fluorescent microscope.

### Hepatocyte culture media

Hepatocyte culture media was prepared with high glucose (4.5 g/L) DMEM (Life Technologies, Cat No. 31600083) supplemented with 10% fetal Bovine Serum (FBS, Hyclone Cat No. SH30071.03), 0.02 mg/L Epidermal Growth Factor (Life Technologies, EGF Cat No. E3476), 0.01428 mg/L Glucagon (Bedford Laboratories, Cat No. 55390-004-01) 7.5 mg/L Hydrocortisone (SOLU-CORTEF® hydrocortisone sodium succinate for injection, Pharmacia Corporation), 500 U/L Insulin (Eli Lily, Cat No. HI-213), 2 mM Glutamine (Life Technologies, Cat No. 15140122) and 2% Penicillin-Streptomycin (Life Technologies, Cat No. 21051040).

### Collagen

Type I Collagen was prepared by extracting acid-soluble collagen from Lewis rat-tail tendons as previously reported^49^. Collagen coating was prepared by mixing collagen with PBS (0.1125 mg/mL). Collagen gel was prepared by mixing collagen (1.125 mg/mL) with 10× DMEM resulting in a solution of ~1 mg/mL Collagen. Freshly prepared gel solution is stored on ice and used immediately.

### Multiple-cell co-culture preparation

#### Hepatocyte only culture

Freshly isolated rat hepatocytes were seeded in collagen coated 12 well plates at 0.5 M cells/well in hepatocyte culture media and incubated overnight at 37 °C, 10% CO_2_. Hepatocytes (after overnight incubation) were washed with PBS and overlaid with 200 μL of collagen gel and incubated for 1 hour at 37 °C, 10% CO_2_. At the end of incubation, 500 μL of hepatocyte maintenance media was added to the well and an empty transwell with 300 μL of media was added to the well.

#### Stellate + (LSEC + Kupffer) culture

(NPC culture) 12 well transwells were coated with 50 μg/mL Fibronectin solution (in PBS) for 1 h at 37 °C. To the coated transwells, 1.0 M LSECs and 0.15 M Kupffer cells were added and incubated overnight. Primary rat stellate cells (in culture) were trypsinized and suspended in freshly prepared collagen gel to a final concentration of 0.25 M/mL. 200 μL of collagen gel with primary stellate cells was added to a collagen coated well and incubated for 1 hour at 37 °C, 10% CO_2_. At the end of incubation, 500 μL of hepatocyte maintenance media was added to the well and a transwell with LSECs and Kupffer cells in 300 μL of media was placed within the well.

#### (Hepatocyte + Stellate) + (LSEC + Kupffer) culture

(Hepatocyte-NPC culture) Freshly isolated rat hepatocytes were seeded in collagen coated 12 well plates at 0.5M cells/well in hepatocyte culture media and incubated overnight at 37 °C, 10% CO_2_. Simultaneously, 12 well transwells were coated with 50 μg/mL Fibronectin solution (in PBS) for 1 h at 37 °C. To the coated transwells, 1.0 M LSECs and 0.15 M Kupffer cells were added and incubated overnight at 37 °C, 10% CO_2_. Primary rat stellate cells (in culture) were trypsinized and suspended in freshly prepared collagen gel to a final concentration of 0.25 M/mL. Hepatocytes (after overnight incubation) were washed with PBS and overlaid with 200 μL of collagen gel containing the primary stellate cells and incubated for 1 hour at 37 °C, 10% CO_2_. At the end of incubation, 500 μL of hepatocyte maintenance media was added to the well and a transwell with LSECs and Kupffer cells in 300 μL of media was placed within the well.

### Albumin Assay

Albumin concentration in the media was evaluated using an in-house ELISA protocol. Briefly, 96-well high-binding ELISA plates were coated with 5 μg/well rat albumin in 100 μL PBS overnight at 4 °C. The plates were washed with PBS-Tween (0.05%) at least 4 times and 50 μL of the media or standards were added to the plate. Each plate has a set of standards. Albumin antibody (http://www4.mpbio.com, Cat No. 55776) was diluted 1:10000 in PBS-Tween and 50 μL was added to each well and incubated overnight at 4 °C or for 2 h at 37 °C. At the end of incubation, the plates were washed with PBS-Tween (0.05%) at least 4 times. A substrate solution of o-Phenylenediamine dihydro-chloride (OPD, 400 μg/mL) and 4 μM H_2_O_2 _solution was prepared in a citric acid buffer. 50 μL of the solution was added to each well and incubated for 5 min. Reaction was stopped by addition of 50 μL 8N H_2_SO_4_ solution and the absorbance was read at 490 nm.

### CYP1A1/2 (EROD) Assay

CYP450 1A/2 activity of the co-cultures was evaluated using 7-ethoxyresorufin. Briefly, cell culture samples were rinsed with Earle’s Balanced Salt Solution (EBSS) 3 times at least with 5 min incubations to remove any phenol red from the media and collagen gel. To each of the wells, 500 μL of substrate (10 μM 7-ethoxyresorufin + 80 μM Dicumarol) was added and incubated at 37 °C. 100 μL of the reagent was withdrawn at 5, 10, 15 and 25 min intervals. Fluorescence from the collected sample was measured at λ_ex_ = 525 ± 10 nm and λ_em_ = 580 ± 10 nm. Rate of resorufin production was calculated by diluting resorufin standard in EBSS.

### TNF-α Assay

TNF-α in the cell media was measured using a BD Biosciences (Cat No. 558535) kit as per the manufacturer’s protocol. Briefly, high-binding ELISA plates were coated with capture antibody (in coating buffer) overnight at 4 °C. The plates were washed with a washing buffer (0.05% PBS-Tween) 3 times and an assay diluent (PBS with 10% FBS) was added to the wells and incubated for 1 hour at RT. At the end of incubation, the plates were washed with PBS-Tween and 100 μL of samples or standards were added to the wells and incubated for 2 hours at RT. The plates were then washed with PBS-Tween followed by addition of detection antibody (in assay diluent) and incubated for 1 hour at RT. At the end of incubation, the plates were washed with PBS-Tween and SAv-HRP (in assay diluent) was added and incubated for 1 hour at RT. The plate was then washed thoroughly with PBS-Tween. To each well, 100 μL TMB substrate was added and incubated for 30 min in dark at RT. The reaction was stopped by addition of 50 μL ELISA stop solution and the absorbance was measured at 450–570 nm.

### IL-10 Assay

IL-10 in the cell media was measured using a BD Biosciences (Cat No. 555134) kit as per the manufacturer’s protocol. Briefly, high-binding ELISA plates were coated with capture antibody (in coating buffer) overnight at 4 °C. The plates were washed with a washing buffer (0.05% PBS-Tween) 3 times and an assay diluent (PBS with 10% FBS) was added to the wells and incubated for 1 hour at RT. Cell culture media samples were diluted 1:1 in assay diluent to ensure the concentration of samples was in range. At the end of incubation, the plates were washed with PBS-Tween and 100 μL of samples or standards were added to the wells and incubated for 2 hours at RT. The plates were then washed with PBS-Tween followed by addition of detection antibody + SAv-HRP (in assay diluent) and incubated for 1 hour at RT. The plate was then washed thoroughly with PBS-Tween. To each well, 100 μL TMB substrate was added and incubated for 30 min protected from light at RT. The reaction was stopped by addition of 50 μL ELISA stop solution and the absorbance was measured at 450–570 nm.

### Lactate Assay

Lactate concentration in media was measured using lactate kit (Trinity Biotech, Cat No. 735-10) using the protocol provided by manufacturer. Briefly, lactate assay reagent was prepared by adding 10 mL distilled water to 1 vial and mixed well. Cell culture media samples were diluted 1:20 (10 μL sample + 190 μL distilled water). 10 μL of the diluted sample media was mixed with 190 μL of the lactate reagent mix and incubated for 10 min in the dark at room temperature. Absorbance of the samples was read at 540 nm.

### Statistical analysis

Data was obtained from n ≥ 3 experiments with n = 2 or 3 samples per condition and averaged. Standard error is plotted for all the conditions.

## Additional Information

**How to cite this article**: Bale, S. S. *et al.* Isolation and co-culture of rat parenchymal and non-parenchymal liver cells to evaluate cellular interactions and response. *Sci. Rep.* **6**, 25329; doi: 10.1038/srep25329 (2016).

## Supplementary Material

Supplementary Information
