# Supplementary material for: Isolation and co-culture of rat parenchymal and non-parenchymal liver cells to evaluate cellular interactions and response
Source: Sci Rep. 2016 May 4;6:25329. doi: 10.1038/srep25329 (PMC4855170; doi:10.1038/srep25329)
Supplement: Supplementary Information [file srep25329-s1.pdf]

Isolation and co-culture of rat parenchymal and non-parenchymal liver cells to evaluate cellular interactions and response

Supplementary Information

Shyam Sundhar Bale [1], Sharon Geerts [1], Rohit Jindal [1], Martin L. Yarmush [1,2,*].

[1] Center for Engineering in Medicine, Massachusetts General Hospital, Harvard Medical School and Shriners Hospital for Children, Boston, MA 02114

[2] Department of Biomedical Engineering, Rutgers University, Piscataway, NJ 08854

* Corresponding author

Prof. Martin L. Yarmush
Center for Engineering in Medicine (CEM)
Massachusetts General Hospital, Harvard Medical School
Shriners Hospital for Children
51 Blossom Street
Boston, MA 02114
E-mail: ireis@sbi.org

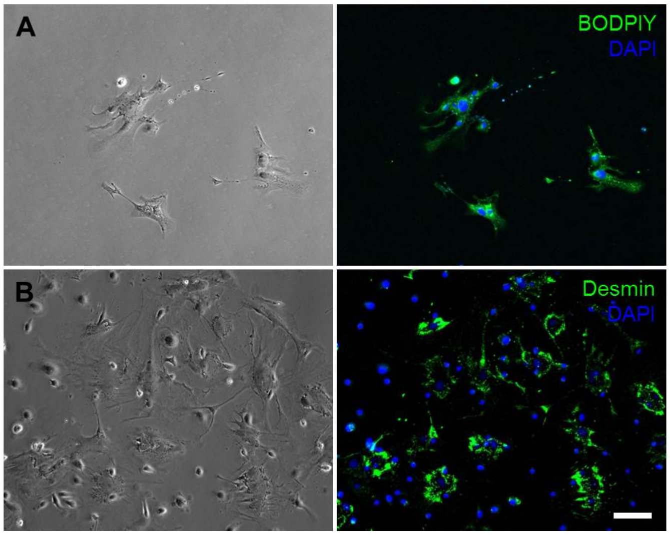

Figure S1. Characterization of stellate cells in culture. Stellate cells in culture (day 7) showing A) Phase and Lipid droplets using BODIPY stain and B) Phase and Desmin stain. Scale bar = 100 μm.

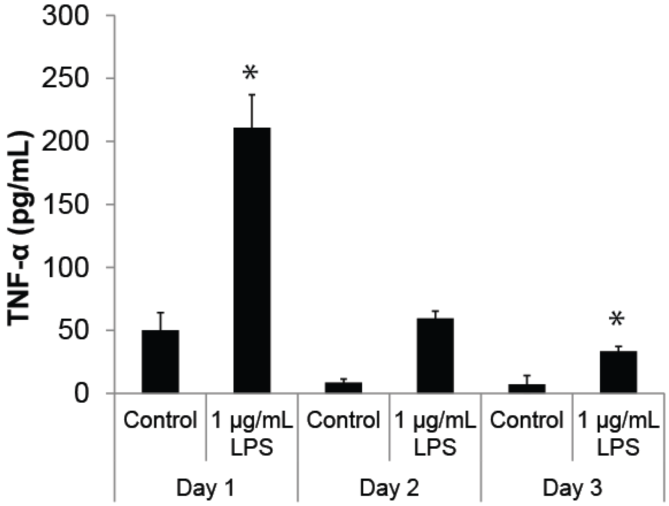

Figure S2. LPS activation and TNF-α response of Kupffer cells in culture. Kupffer cells were cultured in hepatocyte maintenance media and exposed to 1 μg/mL LPS for 24 hours at Day 1, 2 and 3 of culture. (* = $p < 0.05$)

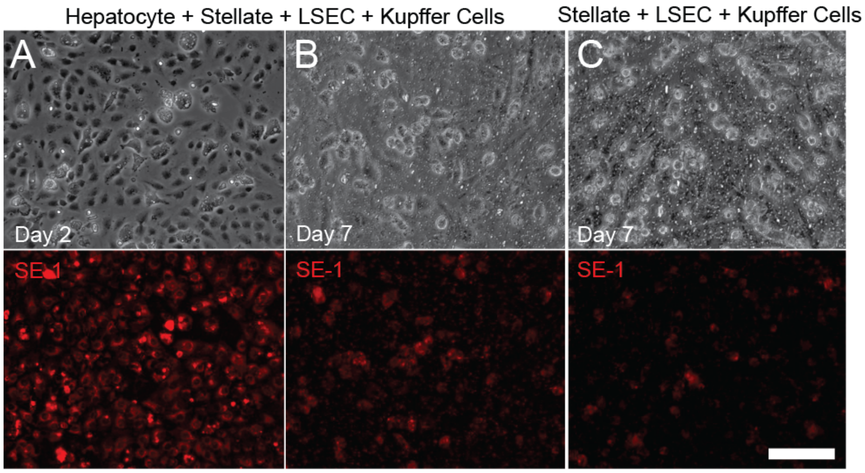

Figure S3. Staining for Endothelial cells at A) Day 2 and B) Day 7 in (Hepatocyte + Stellate) + (LSEC + Kupffer) cultures and C) Day 7 in Stellate + (LSEC + Kupffer) cultures. Scale bar = 100 μm.
